# Complete chloroplast genome and phylogenetic analysis of *Meliosma oldhamii* Miq. ex Maxim. (Sabiaceae)

**DOI:** 10.1080/23802359.2023.2281034

**Published:** 2023-11-27

**Authors:** Yao Cheng, Xiaogang Xu, Lili Tong, Lu Tian, Chongli Xia, Xunlin Yu

**Affiliations:** a College of Biology and the Environment, Key Laboratory of State Forestry and Grassland Administration on Subtropical Forest Biodiversity Conservation, Co-Innovation Center for Sustainable Forestry in Southern China, Nanjing Forestry University, Nanjing, China; b State Environmental Protection Scientific Observation and Research Station for Ecology and Environment of Wuyi Mountains, Nanping, China; c School of Horticulture & Landscape Architecture, Jinling Institute of Technology, Nanjing, China; d College of Forestry, Central South University of Forestry and Technology, Changsha, China

**Keywords:** *Meliosma oldhamii*, complete chloroplast genome, phylogenetic analysis, Sabiaceae

## Abstract

*Meliosma oldhamii* Miq. is a deciduous arbor and a member of Sabiaceae. It is one of the rare and protected plants with outstanding ornamental and economic value in Jiangsu, China. In addition to the resource values indicated above, there are numerous other elements that require additional research, including the chloroplast (cp) genomic information. In this work, the complete cp genome sequence of *M. oldhamii* was assembled and characterized for the first time. The complete cp genome of *M. oldhamii* was 159,950 base pair (bp) in length, including a large single-copy (LSC) region of 87,147 bp, a small single-copy (SSC) region of 18,015 bp, and the inverted repeats (IRs) region of 27,394 bp. It contains 131 genes, including 37 tRNA genes, eight rRNA genes, 85 protein-coding genes, and one pseudogene. The overall GC content of *M. oldhamii* cp genome was 37.95%. The placement of *M. oldhamii* in the phylogenetic tree constructed using the complete cp genome is largely congruent with previous studies. The clustering of *Meliosma* is non-monophyletic, aligns more closely with existing morphological taxonomic studies, thereby enhancing the scholarly and scientific nature of this research.

## Introduction

**Figure 1.**
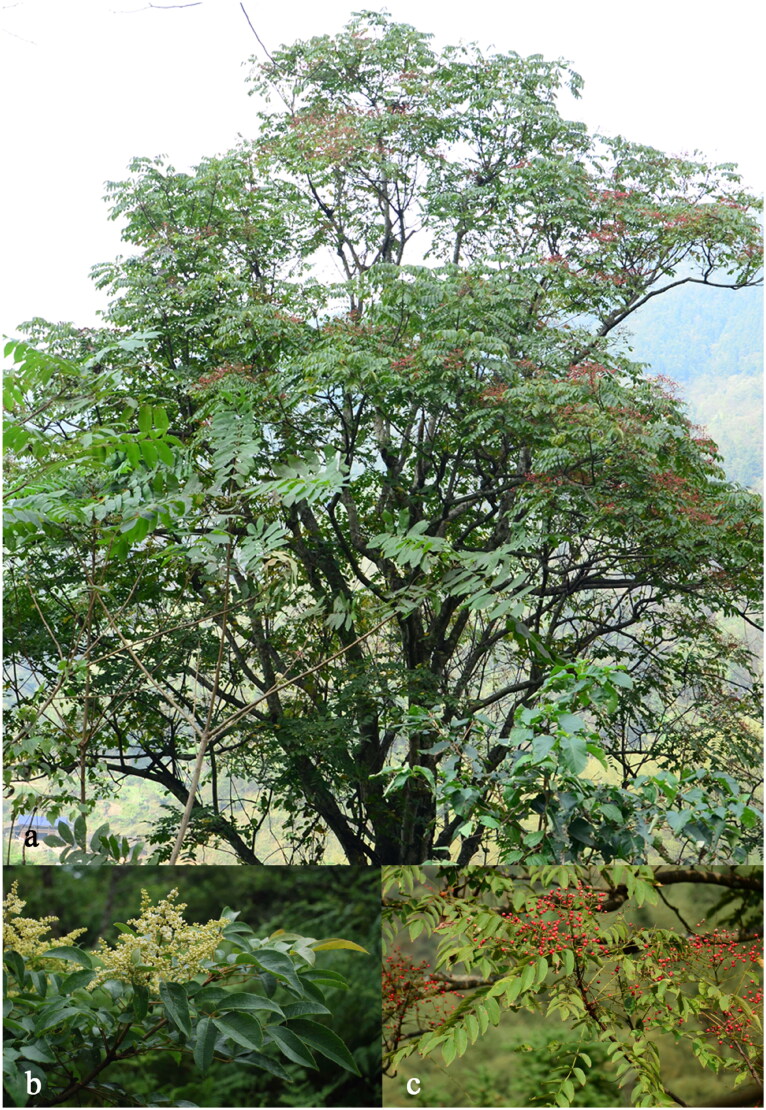
Photo of *Meliosma oldhamii*. (a) The panorama of *M. oldhamii*; (b) the focus on the flowers of *M. oldhamii*; (c) the focus on the fruits of *M. oldhamii* (the photographs of the species reference images were taken by a member of our author team).

*Meliosma oldhamii* (Miquel 1867), a deciduous arbor of Sabiaceae, is one of the rare and protected wild species in Jiangsu, China. It could be up to 20 m tall and mainly distributed in dense and moist valleys and forests at an elevation of 150–1300 m habitats (Figure 1). It is valued for its timber, oil-lubricating ability, and ornamental properties (Guo and Anthony 2007). Previous research has also demonstrated that it may have medicinal properties (Sang and Nam 2015). So far, investigations have concentrated on its community structure study (Cong et al. 2017; Zhu et al. 2020); based on prior research (Zúñiga 2015), the phylogenetic connections in the family Sabiaceae and the genus *Meliosma* need to be ulteriorly confirmed, yet. In this study, the phylogenetic position of *M. oldhamii* in the Sabiaceae was confirmed further by constructing a phylogenetic tree of the complete cp genome. Additionally, the outcome of this work could be a critical step for future conservation, expansion, and utilization.

The objective of this work was to figure out the distinctive characteristics in an attempt to determine the taxonomic placement of the genus *Meliosma*. Here, we characterized the complete cp genome sequence of *M. oldhamii* for the first time based on Illumina pair end sequencing data to provide a valuable complete cp genomic resource, and a new insight into the phylogeny of Sabiaceae.

## Materials

Fresh leaves of *M. oldhamii* were collected from Qixia Mountain Park, in Jiangsu, China (118°57′24″E, 32°9′13″N). The PlantDNA kit was used to extract the total genomic DNA (Genepioneer Biotechnologies, Nanjing, China). The specimens are deposited in the herbarium of Nanjing Forestry University (Xuehong Ma, xuehongma@njfu.edu.cn) under voucher number NF2021099. The Qixia Mountain Scenic Area Administration provided permission for the research, sample collection, and field studies in accordance with the International Union for Conservation of Nature’s (IUCN) policy on endangered species.

## Methods

After passing the sample genomic DNA test, the DNA was fragmented by mechanical interruption (ultrasound), then the fragmented DNA was subjected to fragment purification, end repair, 3′-end addition of A, and ligation of sequencing connectors, followed by agarose gel electrophoresis for fragment size selection and PCR amplification to form sequencing libraries. The constructed libraries were first subjected to library quality control, and the libraries that passed the quality control were sequenced using the Illumina NovaSeq 6000 platform for double-end (PE) sequencing with a read length of 150. Then, filtering of raw data by fastp (version 0.20.0) (Chen et al. 2018) software. The high-quality paired-end reads were assembled through SPAdes (v3.10.1) (Bankevich et al. 2012). The contig sequences obtained were concatenated with SSPACE (v2.0) (Marco and Diego 2018) to obtain scaffolds. The scaffolds were complemented with GAP by Gapfiller (v2.1.1) (Nadalin et al. 2012).

**Figure 2.**
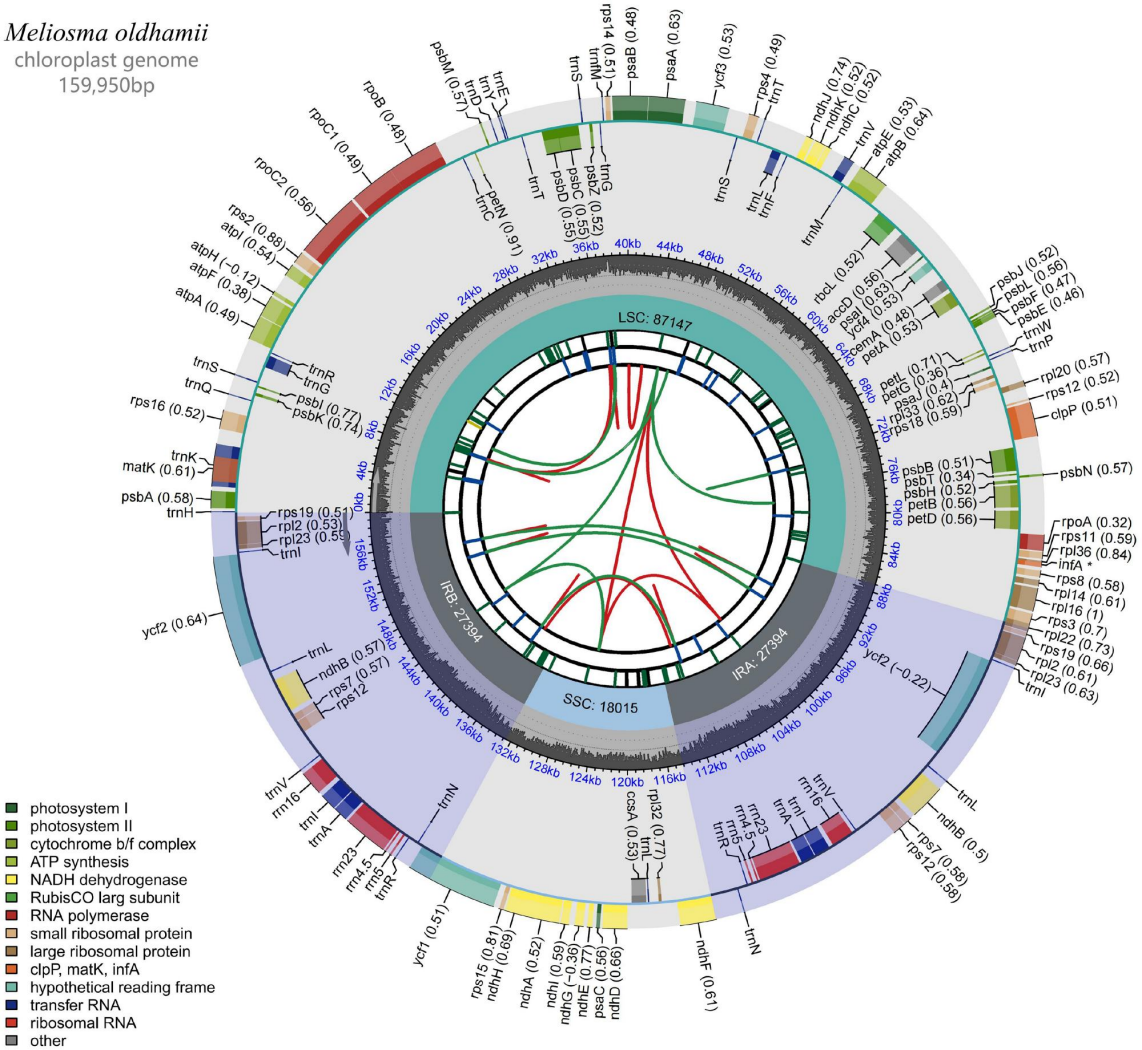
Chloroplast genome map of *M. oldhamii.*

At the same time, the raw data were filtered to remove nodal sequences and low-quality reads to obtain high-quality clean data; the clean data were assembled according to the chloroplast (cp) genome sequence of the reference species to obtain the cp sequence assembly result; the cp sequence assembly result was annotated with the gene structure (reference sequence: *Meliosma* aff. *cuneifolia*, NC_029430.1) and the cp genome was mapped (Figure 2) with CPGView (Liu et al. 2023). Genome coverage map of assembled sequences can be found in supplemental material (Figure S1). The outermost circle represents the genomic sequence; coding genes are represented by green boxes; tRNAs are represented by purple boxes; rRNAs are represented by orange boxes; the innermost green ring represents the depth of coverage; the innermost circle represents the GC content of the genome; segments with >50% GC content are represented by green lines, and vice versa by blue lines. The assembled sequence was registered in NCBI GenBank under the accession number MZ491855.

**Figure 3.**
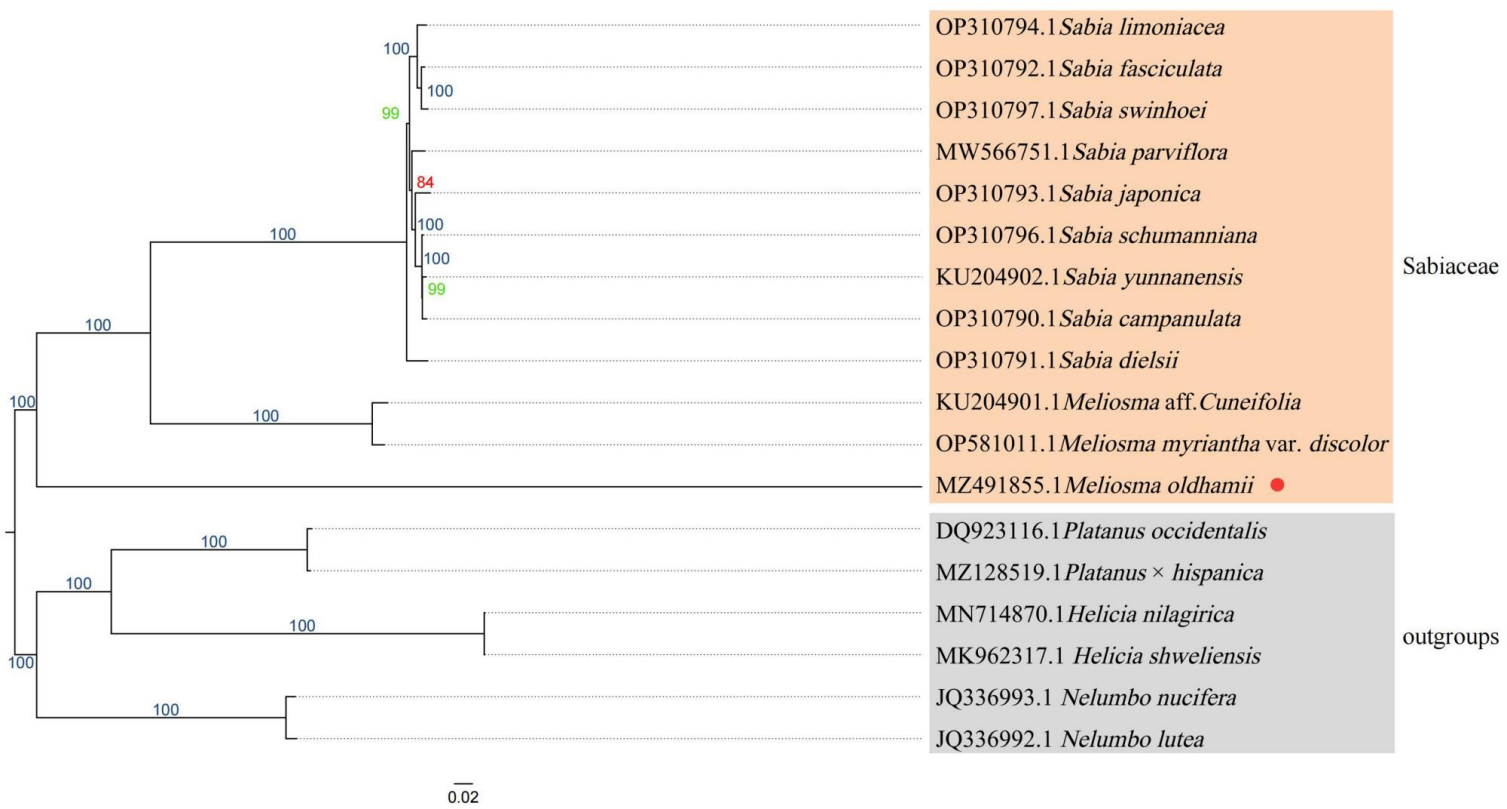
A maximum-likelihood tree was constructed based on the chloroplast genomes of 18 species. The bootstrap supported the values shown at the branches.

To reveal the phylogeny of *M. oldhamii*, the phylogenetic tree (phylogram) was constructed based on the cp genome sequences of Sabiaceae along with the cp genomes of six sequences from Platanaceae, Proteaceae, and *Nelumbo*, respectively, as outgroups (Figure 3). Chloroplast genomes of species closely related to *M. oldhamii* were downloaded from NCBI, and alignment by MAFFT (v7.505) (Rozewicki et al. 2019) and IQTREE (v 2.2.0) (Garg et al. 2021) was used to perform the maximum-likelihood (ML) tree with the TVM + F + I + I + R6 model. Node support was estimated from the results of 1000 bootstrapping replicates.

## Results

The total length of the *M. oldhamii* cp genome sequence was 159,950 bp. The genome had a typical quadripartite structure, including an large single-copy (LSC) region of 87,147 bp and an small single-copy (SSC) region of 18,015 bp separating a pair of inverted repeat (IRa and IRb) sections of 27,394 bp. A total of 131 genes were encoded, including 85 protein-coding genes (78 CDS species), 37 tRNA genes (30 tRNA species), and eight rRNA genes (four rRNA species), and one pseudogene. The majority of the genes were found in a single copy, but seven protein-coding genes (*ndhB*, *rps12*, *rps7*, *rp12*, *rp123*, *rps19*, and *ycf2*), seven tRNA genes (*trnA*-*UGC*, *trnI*-*CAU*, *trnI*-*GAU*, *trnL*-*CAA*, *trnN*-*GUU*, *trnR*-*ACG*, and *trnV*-*GAC*), and four distinct rRNA gene (*23S*, *16S*, *5S*, and *4*.*5S*) are duplicated. A total of 10 protein-coding genes (*atpF*, *ndhA*, *ndhB*, *petB*, *petD*, *rpl16*, *rpl2*, *rpoC1*, *rps12*, and *rps16*) contained one intron while the other two genes (*clpP*, *ycf3*) had two introns each. The cp genome’s total GC concentration was 37.95%. Additionally, the LSC, SSC, and IR areas’ corresponding GC contents were 36.13%, 32.40%, and 42.68%, respectively.

The analysis produced a phylogenetic tree (Figure 3), which showed that *M. oldhamii* forms a separate branch. On the other hand, *M*. aff. *Cuneifolia* and *M. myriantha* var. *discolor* were clustered together into a clade, and the genus *Sabia* formed its own distinct clade. Ultimately, all of these branches were found to be clustered together into the Sabiaceae family.

## Discussion and conclusions

The phylogenetic tree presented in this study (Figure 3) is generally consistent with previous research in Zúñiga (2015), which shows that genus *Sabia* is still monophyletic in Sabiaceae. In Zúñiga’s study, phylogenetic results showed a nonmonophyletic subsect in the genus *Meliosma*, and the object of their work was to suggest the species *M. alba* did not belong to *Meliosma*. In this study, phylogenetic results based on complete cp genome construction can show that *M. oldhamii* belongs to subsect. *Pinnatae* in sect. *Meliosma*, thus reinforcing the framework of Zúñiga’s phylogenetic tree. The overall structure of the cp genome profile of *M. oldhamii* is similar to that of *Meliosma* aff. *cuneifolia* (Figure S2), with differences in gene fragments to be analyzed in further comparisons.

The following conclusions could be drawn from the results: From a morphological perspective, the phylogenetic tree indicated that *M. oldhamii* with a pinnatae feature is a monophyletic clade, distinct from *M*. aff. *Cuneifolia* and *M. myriantha* var. *discolor* with simplices features clustered in another clade.

Given the limited sampling size of the phylogenetic tree reconstructed in this study, it is crucial to gather more relevant sequencing information. As the data of this genus are continuously updated, its phylogenetic status will be clear.

## Supplementary Material

Supplemental Material

## Data Availability

The genome sequence data that support the findings of this study are openly available in GenBank of NCBI at https://www.ncbi.nlm.nih.gov/ under accession no. MZ491855. The associated BioProject, SRA, and Bio-Sample numbers are PRJNA747830, SRR15183923, and SAMN20296681, respectively.
