# Supplementary material for: Complete chloroplast genome and phylogenetic analysis of Meliosma oldhamii Miq. ex Maxim. (Sabiaceae)
Source: Mitochondrial DNA B Resour. 2023 Nov 27;8(11):1306–10. doi: 10.1080/23802359.2023.2281034 (PMC10769540; doi:10.1080/23802359.2023.2281034)
Supplement: Supplemental Material [file TMDN_A_2281034_SM4601.docx]

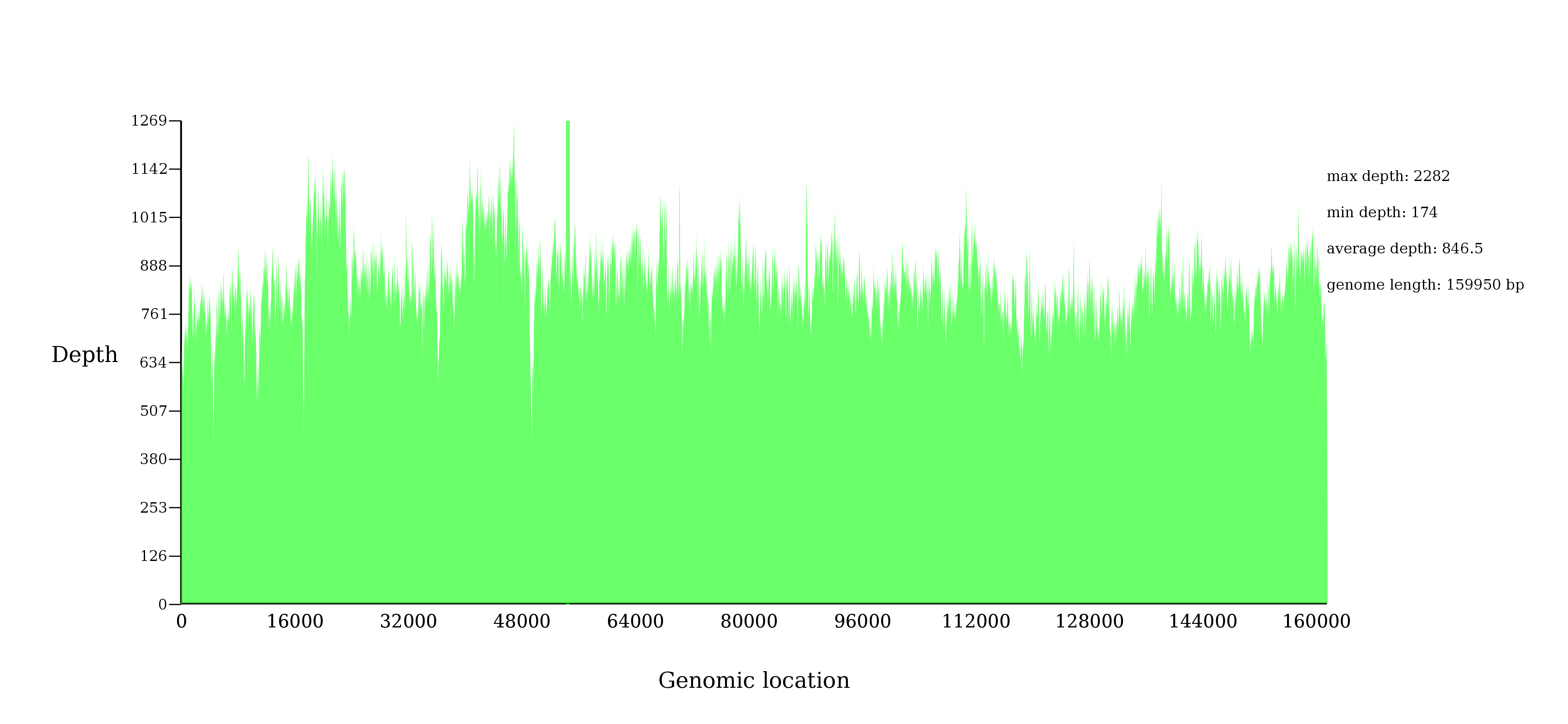


Figure S1. The coverage depth of the complete chloroplast genome of *Meliosma oldhamii.*


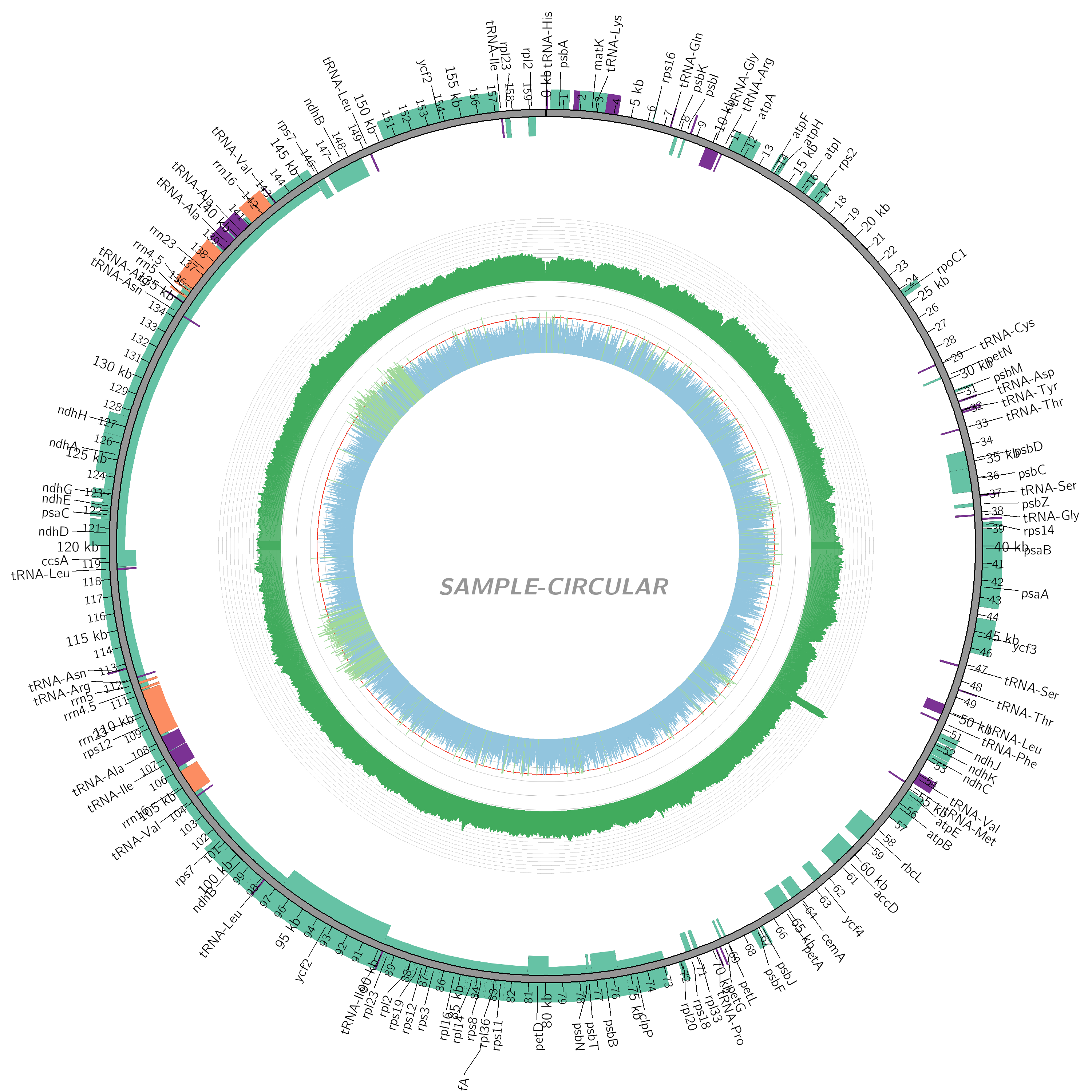


Figure S2. Assembled sequence genome coverage map of the complete chloroplast genome of *M*.*oldhamii*. The outermost circle represents the genomic sequence; coding genes are represented by green boxes; tRNAs are represented by purple boxes; rRNAs are represented by orange boxes; the innermost green ring represents the depth of coverage; the innermost circle represents the GC content of the genome; segments with >50% GC content are represented by green lines, and vice versa by blue lines.


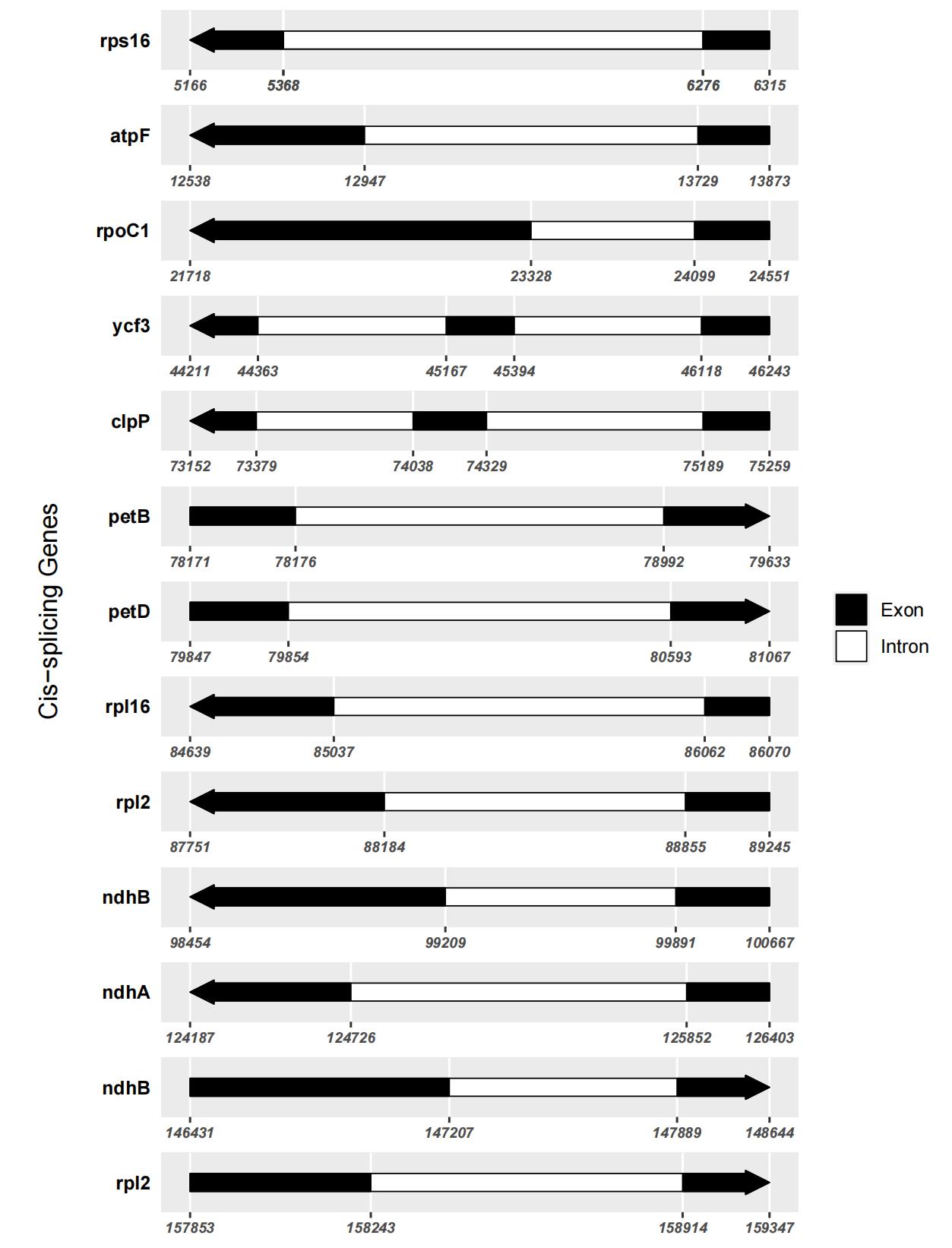


Figure S3. Schematic map of the cis-splicing genes in the chloroplast genome of *M*.*oldhamii*. The genes are arranged from top to bottom based on their order on the chloroplast genome. The exons are shown in black; the introns are shown in white. The arrow indicates the sense direction of the gene.


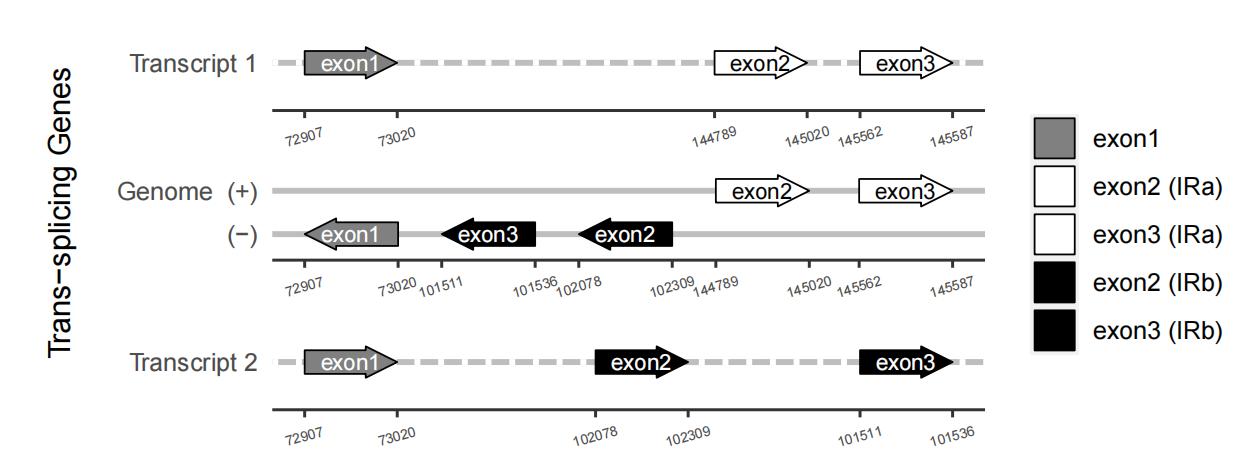


Figure S4. Schematic map of the trans-splicing gene rps12 in the chloroplast genome of *M*.*oldhamii*. It has three unique exons. Two of them are duplicated as they are located in the IR regions.
